# Carboplatin, doxorubicin and etoposide in the treatment of tumours of unknown primary site

**DOI:** 10.1038/sj.bjc.6601785

**Published:** 2004-04-13

**Authors:** A Piga, R Nortilli, G L Cetto, N Cardarelli, S Luzi Fedeli, G Fiorentini, M D'Aprile, F Giorgi, A P Parziale, A Contu, R Montironi, R Gesuita, F Carle, R Cellerino

**Affiliations:** 1 Department of Medical Oncology, University of Ancona, Italy; 2 Department of Medical Oncology, University of, Verona, Italy; 3 Department of Medical Oncology, City Hospital of Pesaro, Italy; 4 Department of Medical Oncology, City Hospital of Ravenna, Italy; 5 Department of Medical Oncology, City Hospital of Latina, Italy; 6 Department of Medical Oncology, City Hospital of San Benedetto, Italy; 7 Department of Medical Oncology, City Hospital of Naples, Italy; 8 Department of Medical Oncology, City Hospital of Sassari, Italy; 9 Department of Pathology, University of Ancona, Italy; 10 Department of Epidemiology and Statistics, University of Ancona, Italy

**Keywords:** carcinoma of unknown primary site, chemotherapy, phase II study

## Abstract

The aim of this study was to assess the activity and toxicity of a platinum-based treatment on a group of patients with unknown primary tumours (UPTs). Patients with a diagnosis of UPT underwent a standard diagnostic procedure. Treatment was started within 2 weeks from diagnosis and consisted of carboplatin 400 mg m^−2^ day 1, doxorubicin 50 mg m^−2^ day 1, etoposide 100 mg m^−2^ days 1–3, every 21 days. Response was evaluated after three courses and treatment continued in case of objective response (OR) or symptom control. A total of 102 patients were eligible. The median age was 59 years, sex male/female 54/48, histology was mainly adenocarcinoma or poorly differentiated carcinoma. Nodes, bone, liver and lung were the most frequently involved sites. In all, 79 patients received at least three courses of treatment; 26 patients received six courses or more. Six complete responses and 21 partial responses were observed, for a total of 27 of 102 ORs or 26.5% (95% confidence interval 18.2–36.1%). The median survival was 9 months and median progression-free survival was 4 months. Toxicity was moderate to severe, with 57.8% of patients experiencing grade III–IV haematological toxicity, mainly leucopenia. The regimen employed has shown activity in tumours of unknown primary site, but was associated with significant toxicity. Such toxicity may be considered unjustified, given the large proportion of patients with tumours not likely to respond. Efforts should therefore be addressed to identify predictors of response to chemotherapy, thus limiting aggressive treatment to those patients who could benefit from it.

Unknown primary tumours (UPTs) are now recognised as an autonomous, although heterogeneous, nosographic entity, with considerable clinical relevance, as they account for 5% of all tumours. Until recently they were approached with more emphasis on diagnosis than on treatment. Much emphasis was placed on trying to ascertain the site of origin of the tumour. This approach is slowly being discarded, at least in reported series, although it widely resists in clinical practice, in particular in non-specialised centres. There are two main reasons for abandoning extensive investigation in an attempt to find the site of origin. Extensive diagnostic procedures cause discomfort for the patient, require time and cause delay of treatment. In addition, they are often fruitless (Hainsworth and Greco, 1993; Abbruzzese *et al*, 1995; Schapira and Jarrett, 1995).

For the minority of tumours that have been identified in the last two decades as being potentially sensitive to chemotherapy (van der Gaast *et al*, 1990; Pavlidis *et al*, 1992; Abbruzzese *et al*, 1995; Lenzi *et al*, 1997; Greco and Hainsworth, 2001b), the diagnostic procedures will delay these patients from receiving effective treatment.

By restricting diagnostic procedures to a minimum, and with an early start of chemotherapy, median survival has improved from 3–6 months of the past (Altman and Cadman, 1986; Alberts *et al*, 1989) to around 1 year in recently reported series (Briasoulis *et al*, 2000; Greco *et al*, 2000a).

We have conducted a multicentre phase II trial in patients with UPT, where diagnostic procedures were limited and where treatment was started soon after presentation. Although in autopsy series the majority of patients with UPT are diagnosed with diseases poorly responsive to treatment (Nystrom *et al*, 1977), there is a substantial minority of patients with primary tumours that are sensitive to chemotherapy, such as germ cell tumours, ovarian and breast cancer. The regimen chosen for this study, a combination of carboplatin, doxorubicin and etoposide, contains drugs active against these more chemosensitive tumours, and employed at dosages potentially able to induce major responses We considered that if an improvement of response rate occurred, this might result in improved outcome for an unselected group of patients with UPTs, and that this would justify the anticipated toxicity of the regimen.

This paper describes the results with emphasis on response to treatment, toxicity and survival.

## PATIENTS AND METHODS

Patients were enrolled in the study if they had a histologically confirmed diagnosis of carcinoma, adenocarcinoma or undifferentiated tumour and no evidence of the site of origin based on routine haematological and biochemical investigation, tumour markers, chest X-ray and abdominal ultrasound. This initial investigation was then completed by CT of abdomen and thorax, and bone scan. Patients with carcinoma or undifferentiated tumour in cervical nodes as the only site of disease were excluded, as they usually deserve specific diagnostic and therapeutic procedures as for head and neck tumours. Other eligibility criteria were: bidimensionally measurable disease and/or elevated tumour markers; age 70 years or less, ECOG performance status ⩽2; and adequate bone marrow (WBC ⩾4000 *μ*l^−1^; platelets ⩾100 000 *μ*l^−1^), renal (creatinine and urea ⩽1.5 × *N*, upper limit of normal) and liver function (bilirubin ⩽1.5 × *N*; liver enzymes <3 × *N*). Patients were excluded if there was a previous diagnosis of cancer at known sites, coexistent cardiac failure or ischaemia, psychiatric disorder or other severe medical illness and less than 3 months of life expectancy.

Following initial workup and assessment of all measurable disease, other diagnostic procedures were those dictated by clinical presentation. The intention was to start treatment within 2 weeks from the diagnosis of UPT.

The pathology workup included immunohistochemistry and, in a limited number of cases, electron microscopy to ascertain epithelial differentiation in some lesions composed of small cells. Immunohistochemistry was carried out on specimens fixed routinely in 10% neutral-buffered formalin for 24 h. Primary antibody was incubated at 4°C for 16–18 h; avidin–biotin–peroxidase complex method was used as a immunodetection method. A variety of antibody reagents were used: cytokeratins – AE1/AE3, CAM 5.2, CK 20, CK 7; epithelial membrane antigen; vimentin; carcinoembryonic antigen; calretinin; S100 protein – placental alkaline phosphatase; thyroglobulin; prostate-specific antigen (PSA); MOC-31; estrogen receptor protein; CA-125; CA 19.9; and tumour-associated glycoprotein (B72.3). Pathology reports were reviewed and classified by one of the authors (RM); no centralised pathology review was carried out.

Patients were treated as outpatients with the following chemotherapy regimen: carboplatin 400 mg m^−2^ day 1, doxorubicin 50 mg m^−2^ day 1 and etoposide 100 mg m^−2^ days 1–3; cycles were repeated every 21 days. At subsequent cycles, if haematological parameters had not recovered by day 22, treatment was delayed for 1 week. Since this was common, most centres adopted a 28-day interval for each cycle. Reduction of doses by 25% was mandatory at the first cycle if patients had advanced age (>65), poor performance status (ECOG 2), multiple organ involvement by the disease, poor renal, cardiac or liver function. This reduction was often maintained throughout all courses based on tolerance. In patients starting with full doses of drugs, a dose reduction of 25% was also planned for subsequent administrations in case of grade 3–4 leucopenia or thrombocytopenia. The use of growth factors on an individual basis was left to the discretion of attending physicians. During therapy, blood counts were not, as a rule, monitored on a weekly basis.

Concomitant antiemetic therapy included 5-hydroxytryptamine-3 antagonists and dexamethasone.

Response was evaluated after three cycles of therapy according to the WHO criteria. Stable and responding patients were subjected to additional cycles based on clinical evaluation. Subsequent treatment in the case of tumour progression at any time was at the discretion of the attending physician.

Response was based on two-dimensional measurement of all sites of disease. Complete response (CR) was complete disappearance of tumour, partial response (PR) reduction of 50% or better of the sum of products of the diameters, stable disease (SD) reduction lower than 50% or less than 25% increase, progressive disease (PD) increase of more than 25% or appearance of new lesions. Survival was calculated from entry in the study till the end of follow-up or death. Progression-free survival was calculated from entry in the study to progression or death from disease (or end of follow-up if not progressed). Toxicity was evaluated according to the WHO criteria (Miller *et al*, 1981).

Statistical evaluation included analysis of survival (Kaplan–Meier), comparison of survival curves (log-rank test) and *χ*^2^ test to assess association between baseline characteristics and toxicity. Carboplatin dosages were converted for statistical purposes to AUC dosing by the Cockroft–Gault (Cockcroft and Gault, 1976) and Calvert (Calvert *et al*, 1989) formulas.

The study was started in January 1991 as a three-institutions study (Ancona, Verona, Pesaro), and was open by the end of the year to the other collaborating centres. Accrual was halted in December 1996; follow-up data were collected on 31st December 2002.

The study was approved by the Ethical Committee of University of Ancona. Written informed consent was requested from patients for entry in the study.

## RESULTS

A total of 113 patients were registered in the study. Of these, 11 patients were judged not eligible: seven because of a previous diagnosis of cancer at known sites, three because they had poorly differentiated carcinoma in cervical lymph nodes as the only site of disease. In one patient, who started treatment before completion of initial workup, abnormal PSA levels led quickly to appropriate diagnostic procedures and to the discovery of the prostatic origin of the tumour. Three patients who exceeded the age limit but who were judged by their physicians to be fit to receive the proposed treatment were included in the analysis.

In all, 102 patients were evaluated; the median age was 59 years (range 25–73 years), male/female ratio of 1.12 (54/48); 51 patients had a performance status ECOG 0, 43 patients ECOG 1, eight patients ECOG 2. Histology was well-differentiated adenocarcinoma (WDA) in 38 cases, poorly differentiated carcinoma or adenocarcinoma (PDC) in 50, squamous cell carcinoma (SCC) in four and undifferentiated neoplasms (UN) in 10 cases. The majority of patients had visceral or bone involvement (60 patients or 58.8%). Eight female patients had peritoneal disease and three patients (two of which male) had disease confined to axillary nodes. Other relevant characteristics of the patients are depicted in Table 1

**Table 1:** Characteristics of the patients (*N*=102)

	**No. of patients**	**%**
*Age (years)*		
Median	59	
Range	25–73	
		
*Sex*		
Male	54	52.9
Female	48	47.1
		
*Performance status*		
ECOG 0	51	50.0
ECOG 1	43	42.2
ECOG 2	8	7.8
		
*Histology*		
WDA	38	37.3
PDC	50	49.0
SCC	4	3.9
UN	10	9.8
		
*Extension*		
Locoregional	28	27.5
Disseminated	74	72.5
		
*Topography*		
Supradiaphragmatic	30	29.4
Subdiaphragmatic	29	28.4
Both sides	43	42.2
		
*Number of involved sites*		
1	29	28.4
2	31	30.4
3	17	16.7
⩾4	25	24.5
		
*Number of metastases*		
1	13	12.7
2	6	5.9
3	12	11.8
⩾4	71	69.6
		
*Main involved sites*		
Supraclavicular nodes	28	27.5
Hylomediastinal nodes	26	25.5
Abdominal nodes	20	19.6
Bone	31	30.4
Liver	27	26.5
Lung	22	21.6
Ascites	12	11.8
Pleural effusion	10	9.8
Nodes/soft tissues only	42	41.2
Visceral/bone involvement	60	58.8
		
*Main symptoms*		
Pain	65	63.7
Gastrointestinal	30	29.4
Respiratory	20	19.6
Fever	16	15.7
Weight loss >10%	9	8.8
		
*Laboratory parameters*		
Hb <12	28	27.5
Any liver index ⩾1.25 × *N*	42	41.2
ALP ⩾1.25 × *N*	21/95	22.1
LDH ⩾1.25 × *N*	26/86	30.2
CEA >5	36/96	37.5
CA 19.9>40	25/84	29.8
CA 125>40	33/69	47.8
Any epithelial marker abnormal	62	60.8
Any germ cell marker abnormal	9	8.8

ECOG=Eastern Cooperative Oncology Group; WDA=well-differentiated adenocarcinoma; PDC=poorly differentiated carcinoma or adenocarcinoma; SCC=squamous cell carcinoma; UN=undifferentiated neoplasms. ALP=alkaline phosphatase; LDH=lactate dehydrogenase; CEA=carcino embrionic antigen; CA 19.9 and CA 125=carbohydrate antigens CA 19.9 and CA 125.

. A total of 74 patients (72.5%) received at least three courses of treatment; 26 patients (25.5%) received six courses of treatment or more.

We observed six CR (5.9%) and 21 PR (20.6%), for a total of 27 of 102 objective responses (OR) or 26.5% (95% confidence interval (CI) 18.2–36.1%), 23 SD (22.5%) and 46 PD (45.1%) (Table 2

**Table 2:** Results of treatment (*N*=102)

	**No. of patients**	**%**
*Responses*		
CR	6	5.9
PR	21	20.6
SD	23	22.5
PD	46	45.1
NA	6	5.9
		
*Duration of response*		
Median (months)	8	
Range	2–102+	
	
*Overall survival*		
Median (months)	9	
At 12 months	35.3%	
At 5 years	6.3%	
	
*Progression-free survival*		
Median (months)	4	
At 12 months	16.3%	
At 5 years	3.6%	
	
*Grade III–IV toxicity*		
Anaemia	31	30.4
Leucopenia	48	47.1
Thrombocytopenia	28	27.5
Nonhaematological toxicity	10	9.8

CR=complete response; PR=partial response; SD=stable disease; PD=progressive disease; NA=not assessable.

). Response was not assessable in six patients (NA, 5.9%). These were patients who died with disease before response could be assessed, and are grouped with nonresponders (intention-to-treat analysis). At the date of last follow-up (December 2002), 94 patients had died. Two of them committed suicide, both with progressing disease.

**Figure 1:**
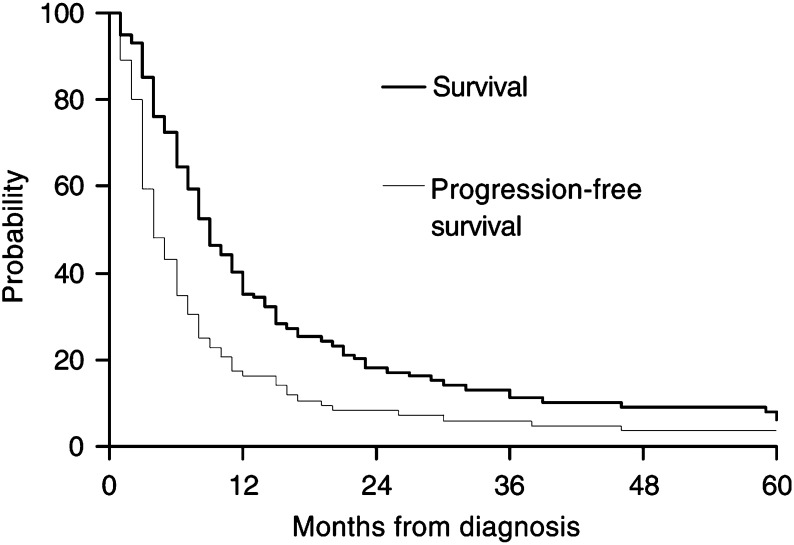
Kaplan–Meier estimates of survival and progression-free survival for the whole group of patients with UPT (*n*=102).

The median survival was 9 months, with 1-year survival of 35.2%, 2-year survival of 18.1%, 5-year survival of 6.3% and median progression-free survival of 4 months (Table2 and Figure 1

).

**Figure 2:**
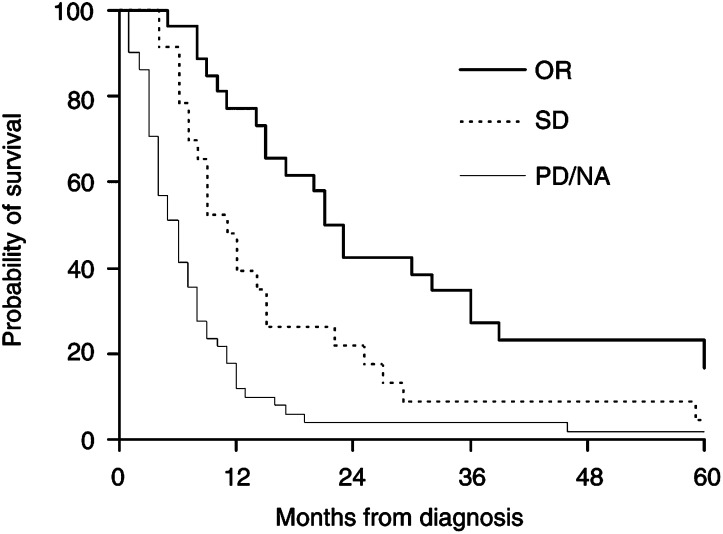
Kaplan–Meier estimates of survival for patients with: (A) OR, *n*=27; (B) SD, *n*=23; and (C) no response (PD/NA, *n*=52). Log-rank test: a *vs* b, *P*=0.008; a *vs* c, *P*<0.001; b *vs* c, *P*=0.003.

The median survival was 23 months for responders, 11 months for SD patients and 6 months for nonresponding patients (Figure 2

). The median duration of response was 8 months.

Toxicity was moderate to severe (Table 3

**Table 3:** Dose intensity and toxicity, overall and by cycle

			**Dose delivered % of projected**				**Delivered dose intensity mg m^−2^ week^−1^ (% of projected)**			**Grade III–IV toxicity number of patients (%)**					
**Cycle no.**	**No. of patients**	**CBDCA AUC median (range)**	**CBDCA**	**DOX**	**VP16**	**Mean interval days**	**CBDCA**	**DOX**	**VP16**	**Overall**	**Haematological**	**Anaemia**	**Leucopenia**	**Thrombocytopenia**	**Gastrointestinal**
1	102	7.8 (2.8–17.2)	85.4	83.2	83.5	26.8	92.8 (69.6)	11.3 (72.0)	68.9 (68.9)	31 (30.4)	28 (27.5)	7 (6.9)	27 (26.5)	9 (8.8)	5 (4.9)
2	85	7.8 (3.0–13.6)	86.3	87.3	86.8	26.8	92.6 (69.5)	11.7 (74.5)	71.0 (71.0)	23 (27.1)	19 (22.4)	8 (9.4)	12 (14.1)	6 (7.1)	4 (4.7)
3	74	7.8 (3.8–13.6)	85.6	85.6	87.3	27.8	90.0 (67.5)	11.3 (72.0)	68.9 (68.9)	19 (25.7)	16 (21.6)	9 (12.2)	15 (20.3)	7 (9.5)	4 (5.4)
4	43	7.7 (3.8–13.6)	83.2	83.8	84.9	27.8	86.7 (65.0)	10.9 (69.4)	66.2 (66.2)	16 (37.2)	15 (34.9)	8 (18.6)	10 (23.3)	6 (14.0)	2 (4.7)
5	34	7.7 (3.8–13.6)	83.5	86.0	85.8	28.9	84.4 (63.3)	10.8 (68.8)	64.8 (64.8)	10 (29.4)	9 (26.5)	6 (17.6)	3 (8.8)	5 (14.7)	1 (2.9)
6	25	7.7 (3.8–13.6)	82.8	84.8	84.8	29.3	83.0 (62.3)	10.6 (67.5)	63.3 (63.3)	3 (12.0)	3 (12.0)	2 (8.0)	1 (4.0)	2 (8.0)	0
															
All	102	7.8 (2.8–17.2)	84.6	84.9	85.2	27.5	89.6 (67.2)	11.2 (71.3)	68.1 (68.1)	64 (62.7)	58 (56.9)	31 (30.4)	48 (47.1)	28 (27.5)	7 (6.9)

CBDCA=carboplatin; CDDP=cisplatin; CYT=cytoxan; DOX=doxorubicin; VP16=etoposide; AUC=area under the curve.

), with 58 patients experiencing grade 3–4 haematological toxicity (mainly leucopenia, but also thrombocytopenia and anaemia) and one patient dying from sepsis during chemotherapy-induced neutropenia. Toxicity other than haematological was limited to occasional gastrointestinal toxicity, while complete reversible alopecia was the rule. One patient had clinically important disturbance of electrolytes and another had transient ECG abnormalities. Details of doses and toxicity are listed in Table 3. Delivered dose intensity, due to either poor general conditions or toxicity, was approximately two-thirds of projected dose intensity (Table 3).

No variable was found associated with toxicity among those assessed, which included: age, sex, performance status, extension of disease, liver involvement, abnormality of liver indexes, calculated AUC for carboplatin and dosage reduction of cytotoxic drugs (Table 4

**Table 4:** Factors examined for the effect on toxicity

**Variable**	**Proportion of patients with grade III–IV toxicity**	***P*-value**
Age ⩽58/>58 years	25/50 (50.0%)/33/52 (63.5%)	0.17
Gender male/female	30/54 (55.6%)/28/48 (58.3%)	0.78
ECOG 0/1–2	29/51 (56.9%)/29/51 (56.9%)	1.00
Locoregional/disseminated disease	14/28 (50.0%)/44/74 (59.5%)	0.39
Disease on one side/both sides of diaphragm	31/59 (52.5%)/27/43 (62.8%)	0.30
One–two metastatic sites/three or more	33/60 (55.0%)/25/42 (59.5%)	0.21
Up to three metastases/four or more	19/31 (61.3%)/39/71 (54.9%)	0.55
No liver involvement/liver involved	46/75 (61.3%) / 12/27 (44.4%)	0.13
No visceral involvement/visceral involvement	26/42 (61.9%)/32/60 (53.3%)	0.39
Normal LDH/abnormal LDH	34/60 (56.7%)/15/26 (57.7%)	0.78
Normal ALP/abnormal ALP	46/74 (62.2%)/9/21 (42.9%)	0.11
Normal liver indexes/abnormal liver indexes	32/59 (54.24%)/25/42 (59.52%)	0.45
No epithelial tumour markers/any marker positive	23/40 (57.5%)/35/62 (56.5%)	0.92
No drug dose reduction/doses reduced	39/70 (55.7%)/19/32 (59.4%)	0.73
CBDCA⩽AUC 8/CBDCA>AUC 8	33/58 (56.9%)/25/44 (56.8%)	0.99
CBDCA⩽AUC 9/CBDCA>AUC 9	39/71 (54.9%)/19/32 (61.3%)	0.55

*Note*: *χ*^2^ test; *P*-values are reported. ECOG=Eastern Cooperative Oncology Group; AUC=area under the curve.

).

## DISCUSSION

The current approach to management of patients with UPT consists of only limited diagnostic investigation followed by an early start of treatment.

With this approach, and with diagnostic and therapeutic improvements (Greco and Hainsworth, 2001b), prognosis seems to have improved to some extent. Response rates to chemotherapy range between 23 and 46% and median survival is between 8 and 11 months (Briasoulis *et al*, 1998a; Culine *et al*, 1999; Briasoulis *et al*, 2000; Greco *et al*, 2001a; Culine *et al*, 2002; Greco *et al*, 2002). It is difficult to compare results of different series, because of the lack of standardised clinical prognostic factors and the limitations of most of the studies, which include small number of patients, variable characterisation of clinical features and short observation period. In general, it appears that more recent chemotherapy regimens that employ platinum compounds, and often etoposide or taxanes or both (Briasoulis *et al*, 1998a; Briasoulis *et al*, 2000; Saghatchian *et al*, 2001; Greco *et al*, 2001a) are superior, in terms of response rate, to more traditional drugs (Kelsen *et al*, 1992; Nole *et al*, 1993; Falkson and Cohen, 1998; Lofts *et al*, 1999).

In the current report, we have treated 102 patients with UPT with an intensive combination of three drugs (carboplatin, doxorubicin and etoposide). A similar combination was used by Briasoulis *et al* (1998a) with lower dosage of carboplatin (300 mg m^−2^) and anthracycline (epirubicin 45 mg m^−2^). We selected these drugs on the basis of their known efficacy in those subsets of UPT that are sensitive to chemotherapy (e.g. germ cell tumours, ovarian carcinomas). We chose to employ these drugs at dosages that might produce as great number of major responses as possible. At these doses toxicity, especially myelosuppression, was expected. The anticipation of toxicity led to restrictive inclusion criteria such as age limit, good general condition and normal organ function, with 92% of patients having ECOG PS of 0 or 1.

We obtained 26.5% of ORs and a median survival of 9 months. Survival at 1 year was 35.3 and 6.3% at 5 years. These results are similar to those of other recently published reports (Table 5

**Table 5:** Results of recent phase II studies in UPT with platinum-based combinations

**Author**	**Publication year**	**No. of patients**	**Chemotherapy**	**Follow-up (months)**	Overall response rate* (%)	**Median survival (months)**	**1-year survival (%)**
Becouarn *et al*	1989	85	CDDP/DOX/5FU/HMM	36 (max)	21.2	7	25
Rigg *et al*	1997	30	CBDCA/5FU/FA	2.8–16.6	26.7	7.8	NA
Falkson and Cohen	1998	40	CDDP/EPI/MIT	NA	50.0	9.4	NA
Briasoulis *et al*	1998a	62	CBDCA/EPI/VP16	40 (max)	37	10	NA
Warner *et al*	1998	33	CBDCA/VP16 os	0.5–33	18.2	5.6	NA
Lofts *et al*	1999	44	CDDP/5FU/TAM	NA	22.7	4	0
Greco *et al*	2000b	71	CBDCA/PTX/VP16 os	34-50	45.1	11	48
Briasoulis *et al*	2000	75	CBDCA/PTX/G-CSF	28 (median)	38.7	13	NA
Greco *et al*	2000a	26	DTX/CDDP	33 (max)	23.1	8	42
		47	DTX/CBDCA	24 (max)	19.1	8	29
Parnis *et al*	2000	43	CDDP/EPI/5FU	24–72	18.6	5.3	NA
Voog *et al*	2000	25	CDDP/VP16	NA	32	8	NA
Dowell *et al*	2001	34	PTX/FA/5FU or CBDCA/VP16	NA	17.6	6.4	26
Saghatchian *et al*	2001	30	CDDP/VP16/IFO/BLM	32 (median)	40	9.4	NA
		18	CDDP/5FU/IFN		44	16	NA
Guardiola *et al*	2001	22	CDDP/DOX/CYT	NA	45.5	10.7	NA
Macdonald *et al*	2002	31	MIT/CDDP/5FU	7–53	27	7.7	28
Culine *et al*	2002	82	CTX/DOX+CDDP/VP16	NA	29.3	10	NA
Greco *et al*	2002	120	CBDCA/PTX/GEM	8–27	23.3	9	42
Present series	2003	102	CBDCA/DOX/VP16	61–120	26.5	9	35.3

*Note*: NA=not available;

* =by intent-to-treat analysis. BLM=bleomycin; CBDCA=carboplatin; CDDP=cisplatin; CYT=cytoxan; DOX=doxorubicin; DTX=docetaxel; EPI=epidoxorubicin; 5FU=5-fluorouracil; FA=folinic acid; GEM=gemcitabine; HMM=hexamethyl-melamine; IFN=alfa-interferon; IFO=ifosfamide; MIT=mitomycin C; PTX=paclitaxel; TAM=tamoxifen; VP16=etoposide; UPT=unknown primary tumours.

), although, as previously indicated, reliable comparison cannot be made between different series.

Toxicity in our patients was moderate to severe. No factor could be identified that was associated with major toxicity. In particular, calculated AUC for carboplatin was not associated with toxicity of chemotherapy. A dose reduction of 25% was routinely applied to patients with advanced age, poor performance status, disseminated disease and poor organ function. This reduction was recommended at first course, but was often maintained through all courses of chemotherapy. It is to be noticed that all patients except two had normal renal function at study entry.

Compared to published series, the regimen we used, employed in an unselected population of patients with UPTs, resulted in no appreciable advantage in terms of response and survival. Toxicity was moderate if compared with the toxicity associated with regimens currently employed in the treatment of chemosensitive tumours; on the other hand, it exceeded the toxicity of regimens employed in tumours where chemotherapy is expected to induce a limited number of responses.

In our view, using an aggressive approach on unselected patients with UPT is not supported by our data and should not be recommended as a routine procedure. Attention should be paid, instead, to the identification of subsets of patients who may benefit from this approach.

Many efforts are now being made in the direction of molecular testing of tumour samples, both as an aid to diagnosis and as an adjunct to available clinical variables that can be used to select groups of patients well defined with regard to prognosis and sensitivity to chemotherapy (Bar-Eli *et al*, 1993; Motzer *et al*, 1995; Pavlidis *et al*, 1995; Briasoulis *et al*, 1998b; Califano *et al*, 1999; Hainsworth *et al*, 2000).

Newer imaging techniques (Tilanus-Linthorst *et al*, 1997; Kole *et al*, 1998; Lenzi *et al*, 1998; Schorn *et al*, 1999; Stevens *et al*, 1999), such as breast MRI, positron emission tomography and other nuclear medicine techniques, that can give clues as to the site of the primary, presently remain of limited help.

There are important psychological aspects of the management of this condition. Two of our patients committed suicide. The failure to identify the site of origin adds to the anxiety and uncertainty of the condition and its treatment. The need for psychological support for these patients is considerable and requires expertise and training in the medical teams.

## Appendix

The following Investigators took part in the trial: A Piga, R Cellerino, N Cardarelli, E Porfiri (Medical Oncology, University of Ancona); R Nortilli, GL Cetto (Medical Oncology, University of Verona); S Luzi Fedeli, V Catalano (Medical Oncology, Pesaro); M D'Aprile, F Cardillo (Medical Oncology, Latina); G Comella, AP Parziale (Medical Oncology, Istituto Scientifico Pascale, Naples); M Marangolo, GM Fiorentini (Medical Oncology, Ravenna); G De Signoribus, F Giorgi (Medical Oncology, San Benedetto del Tronto); N Riva (Medical Oncology, Forlì); P Sandri, G Mustacchi (Medical Oncology, University of Trieste); G Mantovani (Medical Oncology, University of Cagliari); RR Silva (Medical Oncology, Fabriano); S Crispino (Medical Oncology, Arezzo), G Porcile (Medical Oncology, Alba), V Pieroni (Medical Oncology, Jesi); R Montironi (Department of Pathology, University of Ancona); F Carle, R Gesuita (Department of Epidemiology and Statistics, University of Ancona).

## References

[bib1] Abbruzzese JL, Abbruzzese MC, Lenzi R, Hess KR, Raber MN (1995) Analysis of a diagnostic strategy for patients with suspected tumors of unknown origin. J Clin Oncol 13: 2094–2103 7636553 10.1200/JCO.1995.13.8.2094

[bib2] Alberts AS, Falkson G, Falkson HC, van der Merwe MP (1989) Treatment and prognosis of metastatic carcinoma of unknown primary: analysis of 100 patients. Med Pediatr Oncol 17: 188–192 2747591 10.1002/mpo.2950170304

[bib3] Altman C, Cadman E, Lenzi R (1986) An analysis of 1539 patients with unknown primary site. Cancer 57: 120–124 3940611 10.1002/1097-0142(19860101)57:1<120::aid-cncr2820570124>3.0.co;2-m

[bib4] Bar-Eli M, Abbruzzese JL, Lee-Jackson D, Frost P (1993) p53 gene mutation spectrum in human unknown primary tumors. Anticancer Res 13: 1619–1623 8239543

[bib5] Becouarn Y, Brunet R, Barbe-Gaston C (1989) Fluorouracil, doxorubicin, cisplatin and altretamine in the treatment of metastatic carcinoma of unknown primary. Eur J Cancer Clin Oncol 25: 861–865 2500343 10.1016/0277-5379(89)90133-8

[bib6] Briasoulis E, Kalofonos H, Bafaloukos D, Samantas E, Fountzilas G, Xiros N, Skarlos D, Christodoulou C, Kosmidis P, Pavlidas N (2000) Carboplatin plus paclitaxel in unknown primary carcinoma: a phase II Hellenic Cooperative Oncology Group Study. J Clin Oncol 18: 3101–3107 10963638 10.1200/JCO.2000.18.17.3101

[bib7] Briasoulis E, Tsavaris N, Fountzilas G, Athanasiadis A, Kosmidis P, Bafaloukos D, Skarlos D, Samantas E, Pavlidis N (1998a) Combination regimen with carboplatin, epirubicin and etoposide in metastatic carcinomas of unknown primary site: a Hellenic Co-Operative Oncology Group Phase II Study. Oncology 55: 426–430 9732220 10.1159/000011890

[bib8] Briasoulis E, Tsokos M, Fountzilas G, Bafaloukos D, Kosmidis P, Samantas E, Skarlos D, Nicolaides C, Pavlidis N (1998b) Bcl2 and p53 protein expression in metastatic carcinoma of unknown primary origin: biological and clinical implications. A Hellenic Co-operative Oncology Group study. Anticancer Res 18: 1907–1914 9677443

[bib9] Califano J, Westra WH, Koch W, Meininger G, Reed A, Yip L, Boyle JO, Lonardo F, Sidransky D (1999) Unknown primary head and neck squamous cell carcinoma: molecular identification of the site of origin. J Natl Cancer Inst 91: 599–604 10203278 10.1093/jnci/91.7.599

[bib10] Calvert AH, Newell DR, Gumbrell LA, O'Rellly D, Burnell M, Boxall FE, Siddik ZH, Judson IR, Gore ME, Wiltshaw E (1989) Carboplatin dosage: prospective evaluation of a simple formula based on renal function. J Clin Oncol 7: 1748–1756 2681557 10.1200/JCO.1989.7.11.1748

[bib11] Cockcroft DW, Gault MH (1976) Prediction of creatinine clearance from serum creatinine. Nephron 16: 31–41 1244564 10.1159/000180580

[bib12] Culine S, Fabbro M, Ychou M, Romleu G, Cupissol D, Pinguet F (1999) Chemotherapy in carcinomas of unknown primary site: a high-dose intensity policy. Ann Oncol 10: 569–575 10416007 10.1023/a:1026478009050

[bib13] Culine S, Fabbro M, Ychou M, Romleu G, Cupissol D, Pujol H (2002) Alternative bimonthly cycles of doxorubicin, cyclophosphamide, and etoposide, cisplatin with hematopoietic growth factor support in patients with carcinoma of unknown primary site. Cancer 94: 840–846 11857320 10.1002/cncr.10264

[bib14] Dowell JE, Garrett AM, Shyr Y, Johnson DH, Hande KR (2001) A randomized phase II trial in patients with carcinoma of an unknown primary site. Cancer 91: 592–597 11169943 10.1002/1097-0142(20010201)91:3<592::aid-cncr1039>3.0.co;2-5

[bib15] Falkson CI, Cohen GL (1998) Mitomycin C, epirubicin and cisplatin *vs* mitomycin C alone as therapy for carcinoma of unknown primary origin. Oncology 55: 116–121 9499185 10.1159/000011845

[bib16] Greco FA, Burris III HA, Erland JB, Gray JR, Kalman LA, Schreeder MT, Hainsworth JD (2000b) Carcinoma of unknown primary site. Cancer 89: 2655–2660 11135228

[bib17] Greco FA, Burris III HA, Litchy S, Barton JH, Bradof JE, Richards P, Scullin DC Jr, Erland JB, Morrissey LH, Hainsworth JD (2002) Gemcitabine, carboplatin, and paclitaxel for patients with carcinoma of unknown primary site: a Minnie Pearl Cancer Research Network Study. J Clin Oncol 20: 1651–1656 11896116 10.1200/JCO.2002.20.6.1651

[bib18] Greco FA, Erland JB, Morrissey LH, Burris III FA, Hermann RC, Steis R, Thompson D, Gray J, Hainsworth JD (2000a) Carcinoma of unknown primary site: phase II trials with docetaxel plus cisplatin or carboplatin. Ann Oncol 11: 211–215 10761758 10.1023/a:1008369812295

[bib19] Greco FA, Gray J, Burris III HA, Erland JB, Morrissey LH, Hainsworth JD (2001a) Taxane-based chemotherapy for patients with carcinoma of unknown primary site. Cancer J 7: 203–212 11419028

[bib20] Greco FA, Hainsworth JD (2001b) Cancer of unknown primary site. In Cancer: Principles and Practice of Oncology, DeVita JrVT, Hellman S, Rosenburg SA (eds) pp 2537–2560. Philadelphia: Lippincott

[bib21] Guardiola E, Pivot X, Tchicknavorian X, Magne N, Otto J, Thyss A, Schneider M (2001) Combination of cisplatin–doxorubicin–cyclophosphamide in adenocarcinoma of unknown primary site: a phase II trial. Am J Clin Oncol 24: 372–375 11474265 10.1097/00000421-200108000-00012

[bib22] Hainsworth JD, Greco FA (1993) Treatment of patients with cancer of an unknown primary site. N Engl J Med 329: 257–263 8316270 10.1056/NEJM199307223290407

[bib23] Hainsworth JD, Lennington WJ, Greco FA (2000) Overexpression of Her-2 in patients with poorly differentiated carcinoma or poorly differentiated adenocarcinoma of unknown primary site. J Clin Oncol 18: 632–635 10653878 10.1200/JCO.2000.18.3.632

[bib24] Kelsen D, Martin DS, Colofiore J, Sawyer R, Coit D (1992) A phase II trial of biochemical modulation using *N*-phosphonacetyl-L-aspartate, high-dose methotrexate, high-dose 5-fluorouracil, and leucovorin in patients with adenocarcinoma of unknown primary site. Cancer 70: 1988–1992 1381991 10.1002/1097-0142(19921001)70:7<1988::aid-cncr2820700730>3.0.co;2-k

[bib25] Kole AC, Nieweg OE, Pruim J, Hoekstra HJ, Koops HS, Roodenburg JL, Vaalburg W, Vermey A (1998) Detection of unknown occult primary tumors using positron emission tomography. Cancer 82: 1160–1166 9506364 10.1002/(sici)1097-0142(19980315)82:6<1160::aid-cncr22>3.0.co;2-3

[bib26] Lenzi R, Hess KR, Abbruzzese MC, Raber MN, Ordonez NG, Abbruzzese JL (1997) Poorly differentiated carcinoma and poorly differentiated adenocarcinoma of unknown origin: favorable subsets of patients with unknown-primary carcinoma? J Clin Oncol 15: 2056–2066 9164218 10.1200/JCO.1997.15.5.2056

[bib27] Lenzi R, Kim EE, Raber MN, Abbruzzese JL (1998) Detection of primary breast cancer presenting as metastatic carcinoma of unknown primary origin by 111 In-pentetreotide scan. Ann Oncol 9: 213–216 9553668 10.1023/a:1008265113591

[bib28] Lofts FJ, Gogas H, Mansi JL (1999) Management of adenocarcinoma of unknown primary with a 5-fluorouracil–cisplatin chemotherapy regimen (CFTam). Ann Oncol 10: 1389–1392 10631472 10.1023/a:1008309204979

[bib29] Macdonald AG, Nicolson MC, Samuel LM, Hutcheon AW, Ahmed FY (2002) A phase II study of mitomycin C, cisplatin and continuous infusion 5-fluorouracil (MCF) in the treatment of patients with carcinoma of unknown primary site. Br J Cancer 86: 1238–1242 11953879 10.1038/sj.bjc.6600258 PMC2375343

[bib30] Miller AB, Hoogstraten B, Staquet M, Winkler A (1981) Reporting results of cancer treatment. Cancer 47: 207–214 7459811 10.1002/1097-0142(19810101)47:1<207::aid-cncr2820470134>3.0.co;2-6

[bib31] Motzer RJ, Rodriguez E, Reuter VE, Bosl GJ, Mazumdar M, Chaganti RS (1995) Molecular and cytogenetic studies in the diagnosis of patients with poorly differentiated carcinomas of unknown primary site. J Clin Oncol 13: 274–282 7799031 10.1200/JCO.1995.13.1.274

[bib32] Nole F, Colleoni M, Buzzoni R, Bajetta E (1993) Fluorouracil plus folinic acid in metastatic adenocarcinoma of unknown primary site suggestive of a gastrointestinal primary. Tumori 79: 116–118 8346562 10.1177/030089169307900207

[bib33] Nystrom JS, Weiner JM, Heffelfinger-Juttner J, Irwin LE, Bateman JR, Wolf RM (1977) Metastatic and histologic presentations in unknown primary cancer. Semin Oncol 4: 53–58 841350

[bib34] Parnis FX, Olver IN, Kotasek D, Norman J, Taylor A, Russell J, Patterson K, Keefe D, Marafioti T (2000) Phase II study of epirubicin, cisplatin and continuous infusion 5-fluorouracil (ECF) for carcinoma of unknown primary site. Ann Oncol 11: 883–884 10997819 10.1023/a:1008311919633

[bib35] Pavlidis N, Briassoulis E, Bai M, Fountzilas G, Agnantis N (1995) Overexpression of C-myc, Ras and C-erbB-2 oncoproteins in carcinoma of unknown primary origin. Anticancer Res 15: 2563–2567 8669824

[bib36] Pavlidis N, Kosmidis P, Skarlos D, Briassoulis E, Beer M, Theoharis D, Bafaloukos D, Maraveyas A, Fountzilas G (1992) Subsets of tumors responsive to cisplatin or carboplatin combinations in patients with carcinoma of unknown primary site. A Hellenic Cooperative Oncology Group Study. Ann Oncol 3: 631–634 1450045 10.1093/oxfordjournals.annonc.a058290

[bib37] Rigg A, Cunningham D, Gore M, Hill M, O'Brien M, Nicolson M, Chang J, Watson M, Norman A, Hill A, Oates J, Moore H, Ross P (1997) A phase I/II study of leucovorin, carboplatin and 5-fluorouracil (LCF) in patients with carcinoma of unknown primary site or advanced oesophagogastric/pancreatic adenocarcinomas. Br J Cancer 75: 101–105 9000605 10.1038/bjc.1997.16 PMC2222694

[bib38] Saghatchian M, Fizazi K, Borel C, Ducreux M, Ruffie P, Le Chevalier T, Theodore C (2001) Carcinoma of an unknown primary site: a chemotherapy strategy based on histological differentiation – results of a prospective study. Ann Oncol 12: 535–540 11398889 10.1023/a:1011129429499

[bib39] Schapira DV, Jarrett AR (1995) The need to consider survival, outcome, and expense when evaluating and treating patients with unknown primary carcinoma. Arch Intern Med 155: 2050–2054 7575063

[bib40] Schorn C, Fischer U, Luftner-Nagel S, Westerhof JP, Grabbe E (1999) MRI of the breast in patients with metastatic disease of unknown primary. Eur Radiol 9: 470–473 10087118 10.1007/s003300050694

[bib41] Stevens KJ, Smith SL, Denley H, Pinder SE, Evans AJ, Chan SY (1999) Is mammography of value in women with disseminated cancer of unknown origin? Clin Oncol (R Coll Radiol) 11: 90–92 10378633 10.1053/clon.1999.9020

[bib42] Tilanus-Linthorst MM, Obdeijn AL, Bontenbal M, Oudkerk M (1997) MRI in patients with axillary metastases of occult breast carcinoma. Breast Cancer Res Treat 44: 179–182 9232276 10.1023/a:1005774009740

[bib43] van der Gaast A, Verweij J, Henzen-Logmans SC, Rodenburg CJ, Stoter G (1990) Carcinoma of unknown primary: identification of a treatable subset? Ann Oncol 1: 119–122 1706613 10.1093/oxfordjournals.annonc.a057688

[bib44] Voog E, Merrouche Y, Trillet-Lenoir V, Lasset C, Peaud PY, Rebattu P, Negrler S (2000) Multicentric phase II study of cisplatin and etoposide in patients with metastatic carcinoma of unknown primary. Am J Clin Oncol 23: 614–616 11202809 10.1097/00000421-200012000-00018

[bib45] Warner E, Goel R, Chang J, Chow W, Verma S, Dancey J, Franssen E, Dulude H, Girouard M, Correia J, Gallant G (1998) A multicentre phase II study of carboplatin and prolonged oral etoposide in the treatment of cancer of unknown primary site (CUPS). Br J Cancer 77: 2376–2380 9649162 10.1038/bjc.1998.395 PMC2150411

